# Tooth enamel dose coefficients of the ICRP adult mesh-type reference computational phantoms for idealized external neutron exposures

**DOI:** 10.1088/1361-6498/ae6080

**Published:** 2026-05-05

**Authors:** Bangho Shin, Chansoo Choi, Robert J Dawson, Chan Hyeong Kim, Wesley E Bolch

**Affiliations:** 1 J. Crayton Pruitt Family Department of Biomedical Engineering, University of Florida, Gainesville, FL, United States of America; 2 Medical Physics Program College of Medicine, University of Florida, Gainesville, FL, United States of America; 3 Department of Nuclear Engineering, Hanyang University, Seoul, Republic of Korea

**Keywords:** neutron enamel dose coefficients, ICRP reference phantoms, tetrahedral mesh, Monte Carlo simulation, electron paramagnetic resonance (EPR) dosimetry

## Abstract

For application in electron paramagnetic resonance (EPR) dosimetry for neutron fields, the present study establishes a dataset of tooth enamel dose coefficients (DCs) for idealized external neutron exposures using the adult mesh-type reference computational phantoms of the international commission on radiological protection. PHITS Monte Carlo neutron transport simulations were performed to compute DCs for buccal and lingual enamels for the front, front-left, front-right, left, and right teeth for 68 monoenergetic neutrons under antero-posterior (AP), postero-anterior (PA), left-lateral, right-lateral, rotational, and isotropic (ISO) irradiation geometries. The dose contributions from primary neutrons and secondary photons were quantified to account for the respective sensitivities of enamel to neutrons and photons in EPR measurements. The results demonstrated meaningful variation in enamel DCs with irradiation geometry; for example, up to a 5.6-fold difference was observed between the AP and PA geometries for the front lingual enamel. In addition, the results showed that for neutron energies below 20 MeV, secondary photons contributed more than 10% to the total dose, emphasizing the need for separate consideration of primary neutrons and secondary photons when interpreting EPR signals. The neutron enamel DCs established in the present study, in combination with the previously derived photon enamel DCs, will enable improved estimation of individual radiation doses, including organ and effective doses, for realistic mixed neutron-photon exposure scenarios.

## 1. Introduction

Electron paramagnetic resonance (EPR) dosimetry using tooth enamel has been widely recognized as a reliable method for estimating individual radiation doses, particularly in situations where personal dosimeters are unavailable (Romanyukha et al 2000, IAEA 2002, Jacob et al 2002). This technique has been extensively applied in photon dosimetry, where the radiation-induced EPR signal in tooth enamel has been shown to be directly proportional to the absorbed dose over a wide photon energy range (13 keV–1.25 MeV) (Ivannikov et al 2004). To facilitate organ and effective dose estimation based on EPR measurements, several efforts have been made to calculate photon enamel dose coefficients (DCs) using computational human phantoms (Takahashi et al 2001, 2002, 2003, Ulanovsky et al 2005, Shin et al 2026). These studies have provided essential data for converting EPR signals into reliable dose estimates in photon exposure scenarios.

In real-world radiation exposure scenarios, however, neutrons are often present alongside photons, such as in nuclear power reactor accidents or mixed-field occupational exposures. In such cases, relying solely on photon enamel DCs can lead to underestimation of the total absorbed dose, as the contribution from neutrons is not captured. Previous research has demonstrated that neutrons, similar to photons, induce free radicals in tooth enamel (Zdravkova et al 2002, Fattibene et al 2003, Khan et al 2003), although the EPR sensitivity of enamel to neutrons differs quantitatively from that to photons (Bochvar et al 1997, Zdravkova et al 2002, Khan et al 2003, Fattibene et al 2004, 2004, Tikunov et al 2005, Trompier et al 2006, Khailov et al 2007). Recognizing this need, Khailov et al (2010) established enamel DCs for idealized external exposures to neutrons. The DCs were computed using a teeth-defined adult stylized phantom (Cristy and Eckerman 1987, Tolstykh et al 2000), which approximates human anatomy using simplified geometric equations (e.g. spheres, cones, ellipsoids, and toroids).

Recent advancements in computational phantoms have significantly improved anatomical realism, thereby enhancing the reliability of dose estimates (ICRP 2020). In 2021, detailed tooth models were developed based on the micro-computed tomography (CT) images of real human teeth (Shin et al 2021) and were incorporated into the new-generation adult mesh-type reference computational phantoms (MRCPs) of the international commission on radiological protection (ICRP) (ICRP 2020). The MRCPs employ a tetrahedral-mesh structure that allows for smooth surface representation, providing a highly detailed and anatomically accurate depiction of tooth structures. Each tooth, along with its subregions such as enamel, dentin, pulp, and cementum, is individually defined, enabling enamel-specific dose estimates. Leveraging these advancements, Shin et al (2026) recently utilized the MRCPs to establish a comprehensive dataset of enamel DCs for idealized external photon exposures, providing reliable DCs that account for variations in tooth site and enamel regions through a detailed representation of tooth enamel morphology.

Despite these advances, neutron enamel DCs have not yet been established using the ICRP MRCPs, leaving a gap in neutron dosimetry for EPR applications, which limits the accurate reconstruction of doses in mixed radiation fields and reduces the reliability of translating EPR signals into total absorbed dose estimates. The present study aims to address this gap by calculating the neutron enamel DCs for idealized external exposure scenarios using the adult MRCPs implemented in the PHITS Monte Carlo radiation transport code (Sato et al 2024). The DCs were calculated for 68 discrete monoenergetic neutron energies, ranging from 10^−9^ to 10^4^ MeV, across six irradiation geometries—antero-posterior (AP), postero-anterior (PA), left-lateral (LLAT), right-lateral (RLAT), rotational (ROT), and isotropic (ISO)—equivalent to those considered in Yeom et al (2020) that provides the neutron organ and effective DCs based on the MRCPs. Finally, to see the dosimetric impact of the new DCs, the DCs were compared with those from Khailov et al (2010).

## 2. Materials and methods

### 2.1. ICRP-145 adult MRCPs

**Figure 1.**
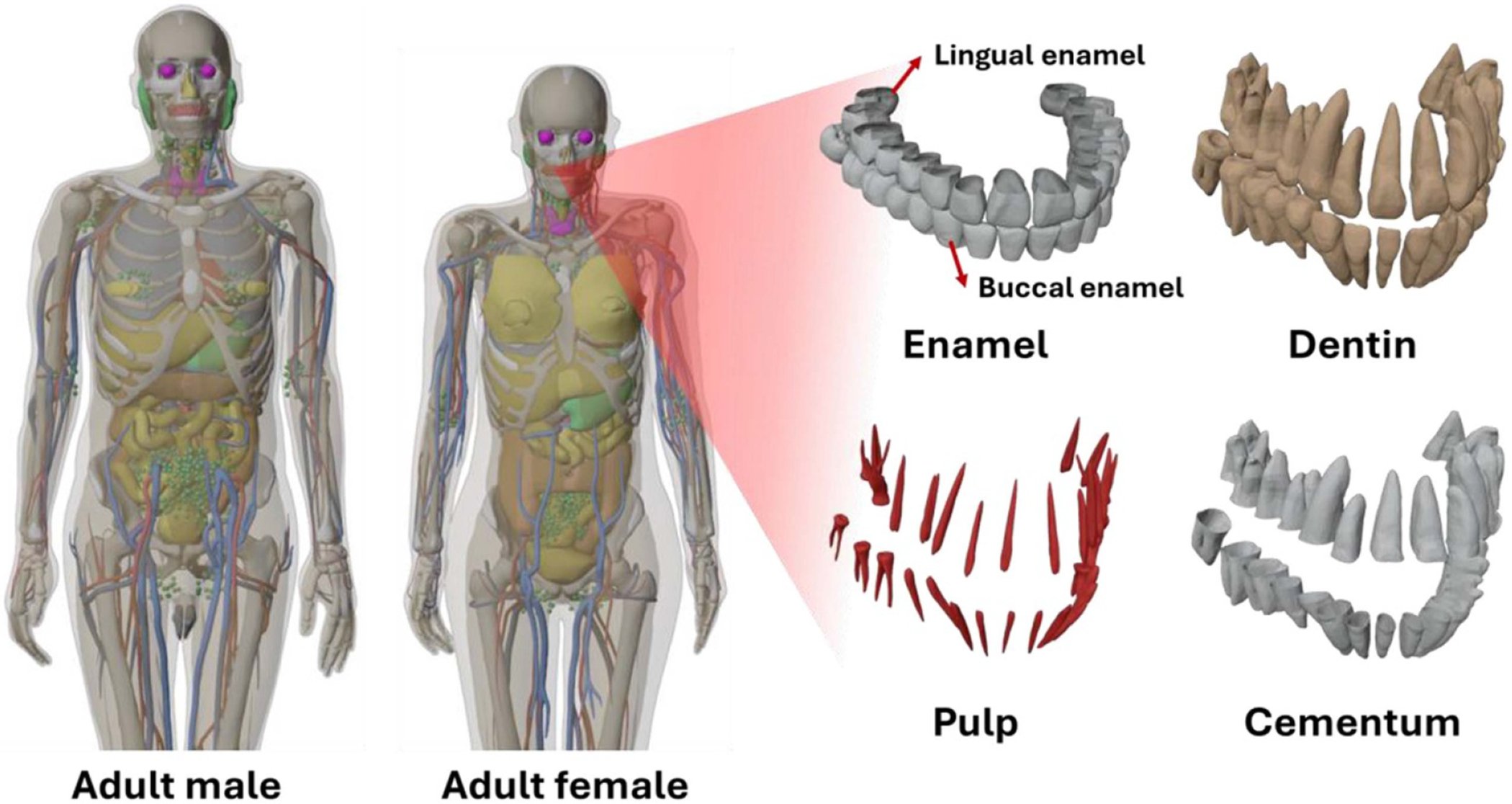
International commission on radiological protection (ICRP) mesh-type reference computational phantoms (MRCPs) for adult male (right) and female (left) and their tooth models.

Figure 1 presents the adult male and female MRCPs utilized in this study (ICRP 2020). These phantoms adhere to the reference anatomical specifications outlined in ICRP Publication 89 (ICRP 2002), with body heights of 176 cm (male) and 163 cm (female) and corresponding body weights of 73 kg (male) and 60 kg (female). The MRCPs define all organs required for effective dose calculations (ICRP 2007). A key feature of these phantoms is the incorporation of highly detailed tooth models for EPR dosimetry applications (Shin et al 2021). The models encompass all 32 permanent teeth, systematically categorized into incisors (central and lateral), canines, premolars (first and second), and molars (first, second, and third) in both the maxillary and mandibular arches. Each tooth is further subdivided into its constituent structures—enamel, dentin, pulp, and cementum—with enamel specifically segmented into buccal and lingual regions. This level of anatomical detail allows for an accurate dose evaluation of the enamel regions predominantly used in EPR signal acquisition, such as the molars, premolars, and the lingual enamel of anterior teeth (IAEA 2002). Constructed in a fourth-generation tetrahedral volume mesh format, the MRCPs phantoms are compatible with general-purpose Monte Carlo simulation codes, including Geant4 (Allison et al 2016), MCNP6 (Goorley et al 2013), PHITS (Sato et al 2024), and EGSnrc (Orok 2022). The total number of tetrahedra is approximately 9.1 million in the male phantom and 9.8 million in the female phantom. Additional details regarding the MRCPs and associated tooth models are available elsewhere (ICRP 2020, Shin et al 2021). Note that the tooth-installed adult MRCPs will be available via the ICRP website in 2026.

### 2.2. Dose calculation cases

**Figure 2.**
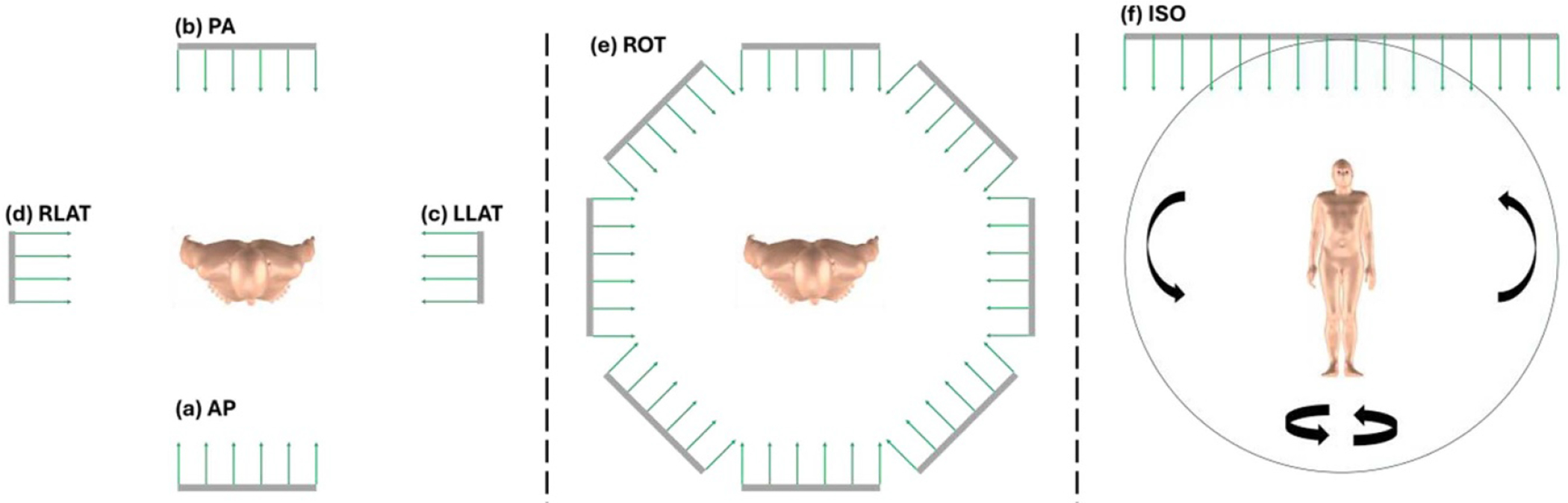
Schematic view of (a) antero-posterior (AP), (b) postero-anterior (PA), (c) left-lateral (LLAT), (d) right-lateral (RLAT), (e) rotational (ROT), and (f) isotropic (ISO) irradiation geometries.

The present study calculated enamel DCs, defined as the absorbed dose averaged over the enamel per unit particle fluence, expressed in pGy cm^2^, for external exposures to neutrons. Following the previous study that established the photon enamel DCs (Shin et al 2026), the enamel was classified into front (incisors and canines), front-left (the left two premolars), front-right (the right two premolars), left (the left three molars), and right (the right three molars) tooth sites, each of which further divided into buccal and lingual regions. The neutron enamel DCs were calculated for broad-parallel beams irradiating the phantoms in six irradiation geometries (i.e. AP, PA, LLAT, RLAT, ROT, and ISO). For each irradiation geometry, the DCs were calculated for 68 monoenergetic neutrons ranging from 10^−9^ to 10^4^ MeV, equivalent to the energy points considered in Yeom et al (2020) that provides the neutron organ and effective DCs of the MRCPs. For AP, PA, LLAT, and RLAT geometries, the phantoms were irradiated by the neutrons emitted orthogonal to the surface of bounding rectangle that fits the phantom size (figures 2(a)–(d)). For ROT geometry, eight phantom-size rectangle sources that emit neutrons perpendicular to their surfaces were placed around the *z*-axis with 45° intervals (figure 2(e)). Finally, for ISO geometry, a circular source of radius 100 cm was uniformly distributed on a spherical shell of radius 100 cm. Neutrons were then emitted perpendicular to the surface of each circular element (figure 2(f)). All irradiations were performed under idealized *in vacuo* conditions.

For neutrons, the EPR dose in enamel can be expressed via the following equation (Khailov et al 2010):

(1) $$D_{\mathrm{EPR}}=D_{\text{E},\gamma }+k_{\text{n}}D_{\text{E},\text{n}}$$

(2) D=∫DC(E)Φ(E)dE

where *D*_EPR_ is the EPR doses in enamel, *D*_E,*γ*_ and *D*_E,n_ are the dose to enamel by the secondary photons and primary neutrons, respectively, *k*_n_ is the EPR sensitivity of enamel to neutrons that can be found in several studies (Bochvar et al 1997, Zdravkova et al 2002, Khan et al 2003, 2004, Fattibene et al 2004, Tikunov et al 2005, Trompier et al 2006, Khailov et al 2007), DC is the DC, and Φ is the particle fluence. The DCs were computed for doses from secondary photons (DC_E,*γ*_) and primary neutrons (DC_E,n_) to calculate *D*_E,*γ*_ and *D*_E,n_, respectively. The total dose, DC_E,T_, was then obtained as the sum of these two contributions. The DC_E,*γ*_ was calculated by scoring the doses to electron and positron, while the DC_E,n_ was calculated by scoring the doses to proton, deuteron, triton, He-3, He-4, nucleus, pion, muon, and kaon, following the relative absorbed dose contribution of secondary particles from the primary neutrons provided in ICRP Publication 116 (ICRP 2010).

### 2.3. PHITS Monte Carlo dose calculations

The enamel DCs were calculated using PHITS Monte Carlo code (version 3.35) (Sato et al 2024). The adult MRCPs, in ‘ELE’ and ‘NODE’ files, were implemented using the ‘LAT = 3’ parameter, which provides the implementation of tetrahedral-mesh geometry. To simulate a parallel-beam exposure scenario, the background material was set to void. The ‘s-type = 2’ parameter for AP, PA, LLAT, RLAT, and ROT geometries and ‘s-type = 9’ for ISO geometry were used in [Source] section to generate monoenergetic neutron beams. The EGS5 algorithm was used to transport photons, electrons, and positrons and the default physics library was used for the other particles. The cut-off energies were set to 1 keV for photons, electrons, and positrons and 10^−5^ eV for neutrons. The event generator mode version 2 was employed using the ‘e-mode = 2’ parameter. The photo-nuclear reaction was considered by ‘ipn-int = 1’ parameter. The thermal scattering data, S(*α, β*), at room temperature (296 K) was employed in transporting the low-energy neutrons by specifying ‘lwtr.20 t’ parameter in [Material] section. The energy depositions in the enamel were scored using [T-deposit] section. The number of primary particles ranged from 10^7^ to 10^10^ depending on the neutron energies and irradiation geometries, keeping the statistical relative errors of the total doses to less than 3%. The simulations were performed on two desktop computers equipped with an Intel^®^ Core^™^ i7-14 700 K processor (8 performance cores, 12 efficient cores, 28 threads, base frequency of 3.4 GHz for performance core, base frequency of 2.5 GHz for efficient core, max turbo frequency of 5.6 GHz) and 64 GB of RAM.

### 3. Results

A comprehensive dataset of neutron enamel DCs was generated for external exposure scenarios using the ICRP-145 adult MRCPs installed with the detailed tooth models coupled with PHITS radiation transport code. The DCs were calculated for the primary neutron and secondary gamma doses, as well as for the total doses, expressed as the absorbed dose averaged over the enamel per unit particle fluence (pGy cm^2^). The dataset provides the DCs for ten distinct enamels: buccal and lingual enamel regions for front, front-left, front-right, left, and right teeth sites. The dataset encompasses 68 discrete monoenergetic energies ranging from 10^−9^ to 10^4^ MeV under six irradiation geometries, i.e. AP, PA, LLAT, RLAT, ROT, and ISO. In total, the dataset includes 24 480 data points. All reported DCs for the total dose exhibit statistical relative errors below 3%. The full neutron enamel DC dataset is available online via GitHub (https://github.com/BH-Shin/JRP-Neutron_enamel_DCs.git).

**Figure 3.**
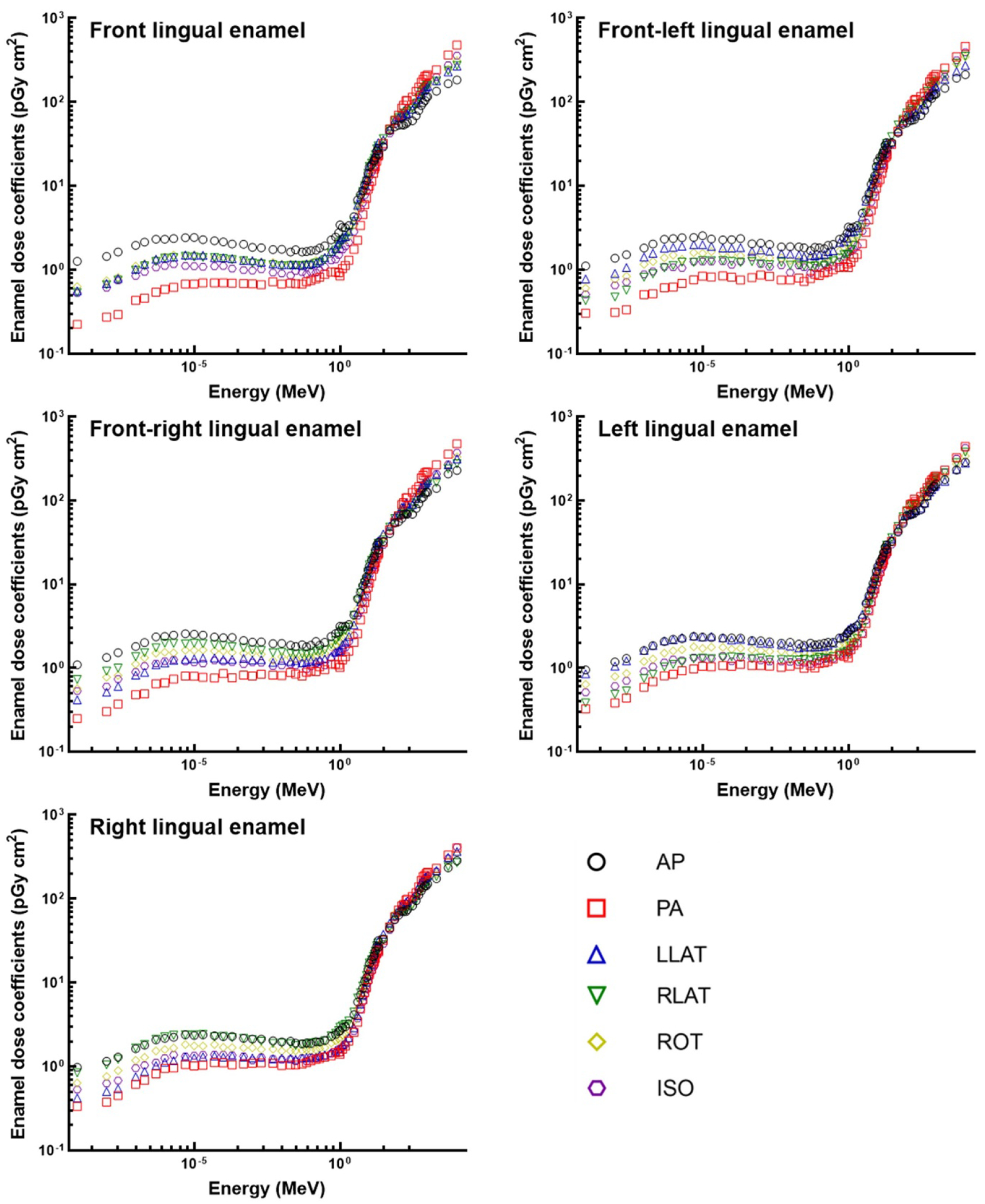
Lingual enamel dose coefficients calculated using adult female mesh-type reference computational phantom in antero-posterior (AP), postero-anterior (PA), left-lateral (LLAT), right-lateral (RLAT), rotational (ROT), and isotropic (ISO) geometries.

Figure 3 shows the lingual enamel DCs for the considered irradiation geometries for female, as examples. It can be seen that for the front lingual enamel, the AP geometry yields the largest values at low energy ranges (<10 MeV), whereas the PA geometry shows the smallest values. The maximum difference is 5.6-fold at 10^−9^ MeV. Conversely, this trend reverses at high energy ranges (>100 MeV). This trend can be explained by the anatomical positioning of the front teeth, which are located close to the lips, resulting in a shallow depth distribution for the AP geometry and a deeper one for the PA geometry. The directional trends are also observed for the other enamel regions. Unlike the front teeth that showed similar DCs for the LLAT and RLAT geometries, for the left-side teeth (front-left and left enamels) and right-side teeth (front-right and right enamels), irradiation geometries incident on the same side (e.g. LLAT for the left teeth) tend to produce higher DCs than those incident on the opposite side (e.g. RLAT for the left teeth) at low energies, whereas the trend reverses at high energies. This trend is owing to the greater depth along the irradiation path for the left- and right-side teeth compared with the front teeth. For the ROT and ISO geometries, the DCs show intermediate values due to the angular averaging effects that mitigate directional dependence.

## 4. Discussion

### 4.1. Comparison of organ DCs with Yeom et al (2020)

**Figure 4.**
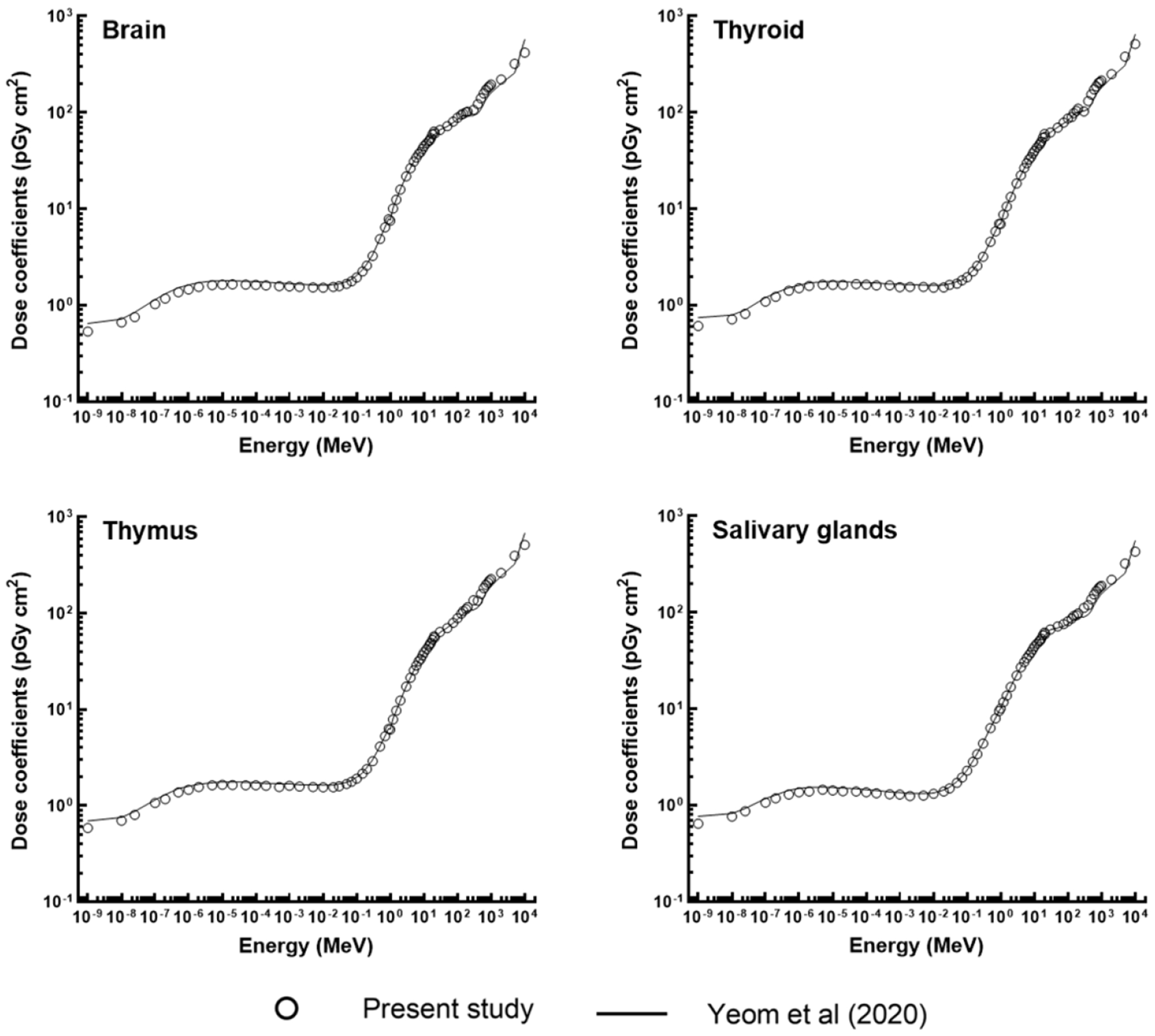
Dose coefficients calculated in present study and those of Yeom et al (2020) for male brain, thyroid, thymus, and salivary glands in isotropic geometry.

Following equations (1) and (2), the enamel DCs can be combined with the organ and effective DCs in Yeom et al (2020) in estimating individual doses in EPR dosimetry, considering that both studies provide DCs for the same phantoms and irradiation geometries. To assess the consistency of dose calculations between two separate studies, we further computed DCs for brain, thyroid, thymus, and salivary glands, which are in close proximity to the teeth, in ISO irradiation geometry, as shown in figure 4. A maximum difference of 31% was observed for the brain at 10 GeV (Yeom et al 2020). These differences are mainly attributed to the use of different physics models and cross sections for particle transport: EGS5, JENDL-4, INCL4.6, JAM, and GEM in PHITS for the present study, versus G4EmLivermorePhysics, G4NeutronHPThermalScattering, G4NeutronHPElastic, G4NeutronHPInelastic, G4NeutronHPCaptureXS, G4NeutronHPFission, and QGSP_BIC_HP in Geant4 for Yeom et al (2020). Minor discrepancies are further explained by subtle modifications to the maxillary and mandibular arches of the cranium and mandible to accommodate the detailed tooth models. Overall, these results demonstrate that the organ DCs derived in this study are consistent with those reported by Yeom et al (2020). Therefore, the enamel DCs presented here can be used in conjunction with their organ and effective DC datasets for individual dose reconstruction.

### 4.2. Dose contributions of primary neutrons and secondary photons

**Figure 5.**
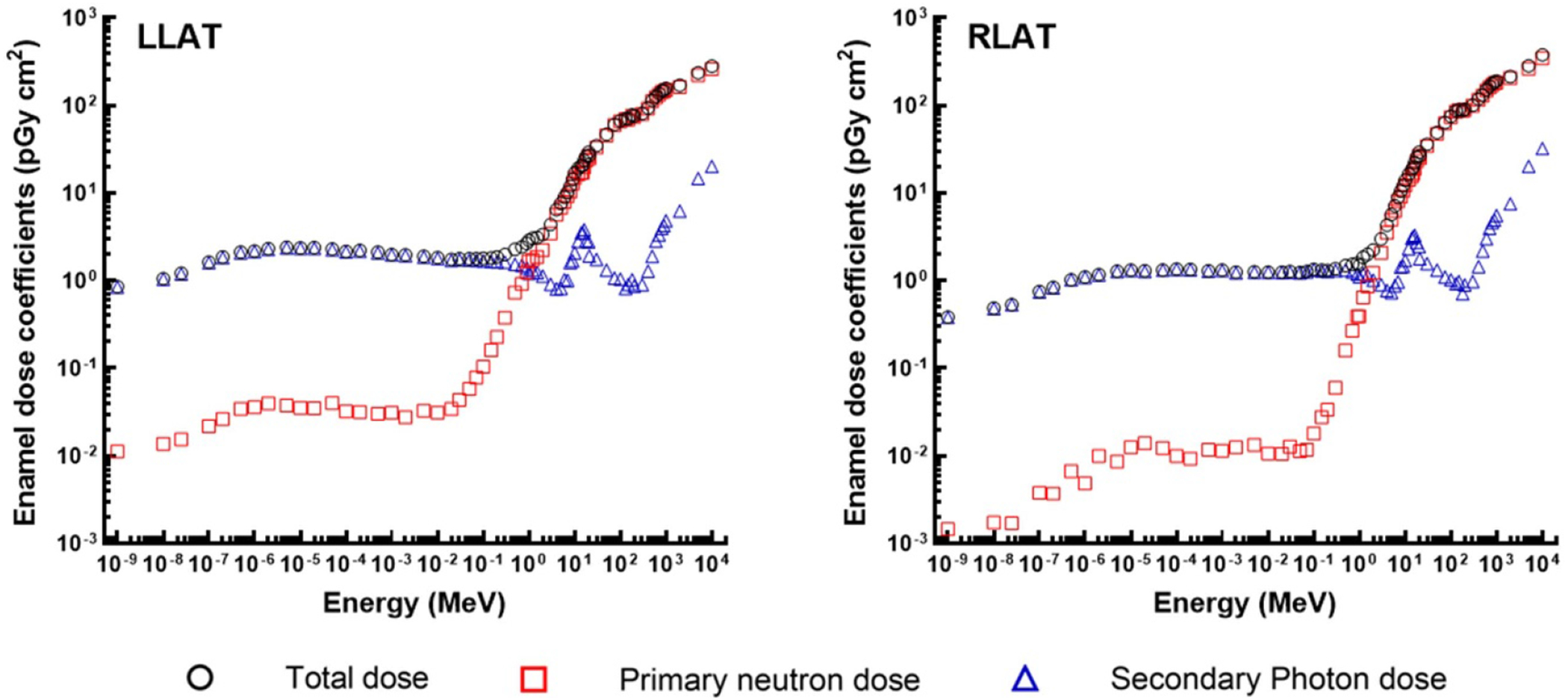
Total, primary neutron, and secondary photon dose coefficients for left lingual enamel calculated using adult female mesh-type reference computational phantom in left-lateral (LLAT) and right-lateral (RLAT) geometries.

In the present study, in addition to the total DCs, the DCs were computed for doses from secondary photons and primary neutrons separately to account for particle-specific EPR sensitivities (equation (2)). The dose contribution of each compartment was investigated for the female left lingual enamel for the LLAT and RLAT geometries, as examples (figure 5). At low energy ranges, the secondary photon contributes major fraction to the total dose. This is mainly because, at low energies, the neutron capture reactions dominantly occur between the neutron and hydrogen and generate gamma rays, resulting in major contribution of secondary photon doses. On the other hand, the contribution of the primary neutron significantly increases with increasing neutron energy above ∼1 keV. At these energies, the recoil protons are generated by elastic scattering, while the charged particles are produced by nuclear reactions at energies above a few MeV. In the tens-to-hundreds of MeV range, inelastic nuclear reactions produce various secondary particles, temporarily lowering the photon contribution to the local enamel dose (ICRP 2010). At even higher energies, increased photon production due to intranuclear cascade processes and meson generation is expected (Bertini et al 1978), potentially leading to a rise in the secondary photon contribution to the enamel dose. Therefore, the contribution of the primary neutron consistently increases with energies, whereas the contribution of the secondary photon exhibits non-monotonic variations. The results indicate that particularly at neutron energies below 20 MeV, secondary photon contributes a significant fraction (exceeding 10%) to the total doses, underscoring the importance of accounting for their contribution when analyzing the EPR signals.

### 4.3. Comparison with Khailov et al (2010)

The dosimetric impact of the established enamel DCs were investigated by comparing the enamel DCs with those given in Khailov et al (2010), which calculated enamel DCs using a teeth-defined adult stylized phantom (Cristy and Eckerman 1987, Tolstykh et al 2000). Table 1 lists the total enamel DCs for the left male enamel. The DCs for the left enamel were obtained as the mass-weighted average of the left buccal and lingual enamel DCs. The comparison was made for neutron energies ranging from 10^−9^ to 20 MeV in AP, LLAT, and RLAT geometries. The DCs are generally in good agreement, showing the ratios of the present study to Khailov et al (2010) from 0.7 to 2.1. These variations may be attributed to a variety of factors, including differences in phantom geometry (e.g. head size), the Monte Carlo codes used, and the physical properties (densities and elemental compositions). For example, the present study used PHITS code and transported the secondary particles, whereas Khailov et al (2010) utilized MCNP4B code and assumed the kerma approximation. At neutron energies below 20 MeV, the kerma approximation is generally considered to provide good dose estimates (ICRP 2010). However, in heterogeneous structures such as the jaws (bones, teeth, and soft tissues), the charged particle equilibrium may not be fully satisfied, potentially introducing dose differences.

Although the enamel DCs obtained using the MRCPs and the stylized phantom are generally comparable, the DCs of the present study show potential dosimetric advantages. While Khailov et al (2010) provided DCs only up to 20 MeV, the present study extends the neutron enamel DCs up to 10^4^ MeV. This enables the dose reconstruction of individuals exposed to space radiations and high-energy accelerators. Furthermore, by employing the most advanced adult MRCPs, the DCs obtained here can be combined with the organ and effective dose DCs in Yeom et al (2020), enabling more precise dose reconstruction. Notably, the MRCPs provide reliable doses by precisely representing human anatomy compared to stylized phantoms, even including microscale radiosensitive target layers (e.g. skin, urinary bladder, and respiratory and alimentary tracts) (ICRP 2020).

## 5. Conclusion

In the present study, we established a comprehensive dataset of neutron enamel DCs using the ICRP-145 adult MRCPs for EPR dosimetry applications. PHITS Monte Carlo simulations were performed for external exposures to 68 monoenergetic neutron energies ranging from 10^−9^ to 10^4^ MeV under AP, PA, LLAT, RLAT, ROT, and ISO irradiation geometries. The results showed that the enamel DCs varied with irradiation geometry; for example, the front lingual enamel DCs for the AP and PA geometries exhibited up to 5.6-fold differences. In addition, it was found that for energies below 20 MeV, secondary photons contributed more than 10% to the total doses, emphasizing the need to account for their contribution when interpreting EPR signals in neutron fields. The neutron enamel DC dataset developed in the present study, in conjunction with the organ and effective DCs of the MRCPs (Yeom et al 2020), will enable improved individual dose estimation, particularly in situations where personal dosimeters are unavailable. Future work will extend this dataset to include photon and neutron enamel DCs for various age groups using the pediatric MRCPs in ICRP Publication 156 (2024).

## Figures and Tables

**Table 1.** Total enamel dose coefficients (pGy cm^2^) for left enamel of male mesh-type reference computational phantom (MRCP) and those of stylized phantom (Khailov et al 2010) in antero-posterior (AP), left-lateral (LLAT), and right-lateral (RLAT) geometries.

Energy (MeV)	Irradiation geometry								
	AP			LLAT			RLAT		
	MRCP	Stylized	Ratio (MRCPs/Stylized)	MRCP	Stylized	Ratio (MRCPs/Stylized)	MRCP	Stylized	Ratio (MRCPs/Stylized)
1.00 × 10^−9^	1.00	1.05	0.95	0.90	1.08	0.83	0.39	0.25	1.54
1.00 × 10^−8^	1.17	1.49	0.79	1.09	1.47	0.74	0.49	0.36	1.36
1.00 × 10^−7^	1.73	2.22	0.78	1.69	2.25	0.75	0.77	0.50	1.54
1.00 × 10^−6^	2.32	2.51	0.92	2.30	2.93	0.78	1.13	0.57	1.98
1.00 × 10^−5^	2.44	2.47	0.99	2.42	2.13	1.14	1.36	0.77	1.77
1.00 × 10^−4^	2.31	2.04	1.13	2.30	1.93	1.19	1.32	0.67	1.97
1.00 × 10^−3^	2.18	1.88	1.16	2.12	1.77	1.20	1.30	0.63	2.06
1.00 × 10^−2^	1.92	1.85	1.04	1.89	1.37	1.38	1.26	0.76	1.66
1.00 × 10^−1^	2.74	2.45	1.12	2.91	2.53	1.15	1.45	0.96	1.51
1.00 × 10^0^	2.74	3.33	0.82	2.91	3.47	0.84	1.45	0.88	1.65
2.00 × 10^0^	3.37	3.72	0.91	3.41	3.68	0.93	2.13	1.76	1.21
4.00 × 10^0^	6.23	6.88	0.91	6.54	6.94	0.94	3.92	2.98	1.32
6.00 × 10^0^	8.95	10.50	0.85	9.34	10.10	0.92	6.26	5.14	1.22
8.00 × 10^0^	12.00	12.60	0.95	12.20	12.50	0.98	8.92	7.53	1.18
1.00 × 10^−1^	17.20	17.00	1.01	17.80	16.40	1.09	12.40	9.74	1.27
1.20 × 10^−1^	19.20	19.70	0.97	20.00	19.40	1.03	14.40	11.80	1.22
1.40 × 10^−1^	21.00	21.90	0.96	22.20	22.10	1.00	16.10	13.90	1.16
1.60 × 10^−1^	23.90	23.60	1.01	26.10	23.00	1.13	19.00	15.20	1.25
1.80 × 10^−1^	27.60	24.10	1.15	30.30	24.20	1.25	22.00	16.40	1.34
2.00 × 10^−1^	30.10	26.40	1.14	34.00	26.60	1.28	24.80	18.40	1.35

## Data Availability

The complete neutron enamel dose coefficients dataset is provided via a GitHub repository available online (https://github.com/BH-Shin/JRP-Neutron_enamel_DCs.git).
